# Addressing Non-linear System Dynamics of Single-Strand RNA Virus–Host Interaction

**DOI:** 10.3389/fmicb.2020.600254

**Published:** 2021-01-15

**Authors:** Alessandra Romano, Marco Casazza, Francesco Gonella

**Affiliations:** ^1^Sezione di Ematologia, Dipartimento di Chirurgia Generale e Specialità Medico Chirurgiche (CHIRMED), Università degli Studi di Catania, Catania, Italy; ^2^Division of Hematology, U.O.C di Ematologia, Azienda Ospedaliero Universitaria Policlinico “G.Rodolico - San Marco”, Catania, Italy; ^3^Dipartimento di Scienze Molecolari e Nanosistemi, Università Ca’ Foscari Venezia, Venezia, Italy

**Keywords:** systems thinking (ST), RNA-virus, virus–host interaction, dynamics, modeling, simulation – computers, evolution trajectories

## Abstract

Positive single-strand ribonucleic acid [(+)ssRNA] viruses can cause multiple outbreaks, for which comprehensive tailored therapeutic strategies are still missing. Virus and host cell dynamics are tightly connected, generating a complex dynamics that conveys in virion assembly to ensure virus spread in the body. Starting from the knowledge of relevant processes in (+ss)RNA virus replication, transcription, translation, virions budding and shedding, and their respective energy costs, we built up a systems thinking (ST)–based diagram of the virus–host interaction, comprehensive of stocks, flows, and processes as well-described in literature. In ST approach, stocks and flows are expressed by a proxy of the energy embedded and transmitted, respectively, whereas processes are referred to the energy required for the system functioning. In this perspective, healthiness is just a particular configuration, in which stocks relevant for the system (equivalent but not limited to proteins, RNA, DNA, and all metabolites required for the survival) are constant, and the system behavior is stationary. At time of infection, the presence of additional stocks (e.g., viral protein and RNA and all metabolites required for virion assembly and spread) confers a complex network of feedbacks leading to new configurations, which can evolve to maximize the virions stock, thus changing the system structure, output, and purpose. The dynamic trajectories will evolve to achieve a new stationary status, a phenomenon described in microbiology as integration and symbiosis when the system is resilient enough to the changes, or the system may stop functioning and die. Application of external driving forces, acting on processes, can affect the dynamic trajectories adding a further degree of complexity, which can be captured by ST approach, used to address these new configurations. Investigation of system configurations in response to external driving forces acting is developed by computational analysis based on ST diagrams, with the aim at designing novel therapeutic approaches.

## Introduction

Positive single-stranded ribonucleic acid [(+)ssRNA] viruses, including picornaviruses, flaviviruses, Togaviridae, and human coronaviruses (CoVs) (Ahlquist et al., 2003; Lam et al., 2016; Scutigliani and Kikkert, 2017; Primadharsini et al., 2019), cause multiple outbreaks, for which tailored antiviral strategies are still missing (Zumla et al., 2016; Dinesh et al., 2020; Gordon et al., 2020b). (+)ssRNA viruses package their genomes as messenger sense, single-stranded RNA and replicate those genomes solely through RNA intermediates in the cytosol of the host cells (Den Boon et al., 2010). RNA-dependent RNA polymerases lack coreplicative and postreplicative fidelity-enhancing pathways; this final RNA genome copies incorporate mutations at a high rate (Lauring et al., 2013; Acevedo et al., 2014), providing the viral quasi-species with a higher probability to evolve and adapt to new environments and challenges during infection (Burch and Chao, 2000; Vignuzzi et al., 2006). The diversity is essential for both viral fitness (Wargo and Kurath, 2012) and pathogenesis because of the complex relationships among virus replication (VR), host cells, and immune system, as almost all (+)ssRNA viruses can delay antiviral innate immune response (Kobasa et al., 2007) in multiple ways (Diebold et al., 2003; Hogan et al., 2004; Meylan et al., 2005; Saito et al., 2008; Yang et al., 2015; Beachboard and Horner, 2016; Nelemans and Kikkert, 2019). Host immunogenetic factors can be sensitive to a variation in the viral load, leading to a defective response of the innate immunity, that could explain the variable clinical course of infection (Fanning et al., 2001; Nelemans and Kikkert, 2019).

Recent studies confirmed the complexity of viral dynamics, whose fitness is improved by the complex interactions with the host proteins (Bosl et al., 2019; Sruthi and Prakash, 2019), as previously described in modeling the virus–host interactions at subcell and cell levels (Dapat and Oshitani, 2016; Jonsdottir and Dijkman, 2016; Gao et al., 2017; Patzina et al., 2017; Gordon et al., 2020b). However, models that address a specific aspect of the virus–host interaction do not capture the wide range of intertwined spatial and temporal (hours to days) dynamic scales (Apweiler et al., 2018), which are related to the interactions of different concurrent hierarchical levels. For this reason, we aimed at describing the virus–host cell interaction as a dynamic system by a systems thinking (ST) approach (Northridge and Metcalf, 2016).

In ST, the behavior of a dynamic system can be described and predicted by the temporal evolution of its configurations, given by hierarchical feedback loops and self-organization. The configuration evolution then can be analytically computed by proper simulators (Odum and Odum, 2000; Tegner et al., 2009; Hassmiller Lich et al., 2017; Spill et al., 2018) to further address suitable leverage points for intervening (Meadows and Wright, 2008). In particular, dynamic models, where the temporal evolution of extensive variables (stocks) is simulated in the form of trajectories, derive their initial conditions from available and assessed evidence. Varying the key system parameters, trajectories represent the possible evolutive (structural) patterns of the system at issue, becoming abstracted with respect to local specific attributes related to single case studies. However, being stocks associated to system observables, the relation with those attributes is maintained, making it possible to compare predicted trajectories with observed data and constituting suitable counterfactuals with respect to the laboratory measurements results.

System dynamics (SD) approach is mostly used for strategic modeling, typically for ecological and socioeconomic systems, to understand the supply chain performance. In particular, results of SD modeling provide a set of alternative evolutive patterns in form of graphs, capturing the internal dynamics of a system even in lack of some experimental data to fit. These can provide alternative scenarios that, when fitting experimental evidences, indicate the most effective leverage points to control the system evolution.

In this article, we show that, by approaching the host–virus interaction as a dynamic systemic problem (Sterman, 2002; Meadows and Wright, 2008), it is possible to identify potential systemic leverage points to minimize the release of virions, so addressing effective *systemic* intervention strategies.

## Materials and Methods

### Development of a Stock-Flow Diagram

The basic SD element is the stock and flow diagram. Stocks are countable extensive variables *Q*_i_, *i* = 1, 2,…, *n*, relevant to the study at issue, that constitute an *n*-ple of numbers (possibly derived from experimental measurements), which at any time represents the state of the system. A stock may change its value only upon its inflows and/or its outflows, represented by arrows entering or exiting the stock. Processes are any occurrence capable to alter—either quantitatively or qualitatively—a flow, by the action of one or more of the system elements. In a stationary state of the system, stock values are either constant or regularly oscillating. Processes, which cause the stationarity or perturbation of a system, must be activated by a driver, acting on the flows where the process is located. The pattern of the feedbacks acting in the system configurations is the feature that ultimately defines the systems dynamics. Each flow depends on the state variables *Q*_i_ by relationships of the kind *dQ*_i_/dt = *kf*(*Q*_*j*_), *i*, *j* = 1,…, *n*, where *n* is the number of stocks in the system.

The stocks and flows inventory reported in Table 1 was based on information from existing knowledge on the biological mechanisms at issue, listing the variables and parameters necessary to set up the equations describing the system dynamics. Turnover times of stocks included in the RNA-virus–host interaction ST diagram have been reported in Table 2, derived from the available literature (Konig et al., 2008; Friedel and Haas, 2011; Pfefferle et al., 2011; Jourdan et al., 2012; Munday et al., 2012; Naji et al., 2012; Wu et al., 2012; Emmott et al., 2013; Garcia-Dorival et al., 2014; Verchot, 2014; Watanabe et al., 2014; York et al., 2014; Zheng et al., 2014; Dong et al., 2016; Kuo et al., 2016, 2018; Wang et al., 2016, 2017a, b; Gao et al., 2017; Hafirassou et al., 2017; Khamina et al., 2017; King et al., 2017; Martinez-Gil et al., 2017; Patzina et al., 2017; Coyaud et al., 2018; Iwasaki et al., 2018; Lescar et al., 2018; Ziegler et al., 2018; Bosl et al., 2019; Chakravorty et al., 2019; Garcia-Moreno et al., 2019; Rothenburg and Brennan, 2020). All stocks, flows, and processes were expressed using a common proxy unit, representing the energy embedded, transmitted, and used, respectively, during the system operation. The proxy unit was expressed as the number of ATP (and ATP-equivalent) hydrolysis events (Mahmoudabadi et al., 2017). This choice allowed calculating each parameter of the system on the basis of stocks and characteristic times of the flows derived from the literature, without further need for experimental data.

**TABLE 1:** Inventory of stocks and flows depicted in the diagram of Figure 1.

Stock	Biological meaning	Dynamic equation*	Calibration value	References
*Q*_1_	Resources available for protein synthesis	d*Q*_1_/dt = *J*_0_ + *J*_21__*A*_ + *J*_21__*B*_ − *J*_1_ − *J*_13_ − *J*_15_ − *J*_17_	3.9	Odum, 1996, 2002; Odum and Odum, 2000
*Q*_2__*A*_	Short-half-life proteins	d*Q*_2__*A*_/dt = *J*_2__*A*_ − *J*_21__*A*_ − *J*_20__*A*_	13	Wheatley et al., 1980; Eden et al., 2011; Siwiak and Zielenkiewicz, 2013
*Q*_2__*B*_	Long-half-life proteins	d*Q*_2__*B*_/dt = *J*_2__*B*_ − *J*_21__*B*_ − *J*_23_ − *J*_25_ − *J*_27_ − *J*_20__*B*_	13	Wheatley et al., 1980; Eden et al., 2011; Siwiak and Zielenkiewicz, 2013
*Q*_3_	Viral ss + RNA	d*Q*_3_/dt = *J*_3_ − *J*_4_	3	Baccam et al., 2006
*Q*_4_	Viral proteins	d*Q*_4_/dt = *J*_4_ − *J*_5_	0.024	Kummer et al., 2014
*Q*_5_	Virions	d*Q*_5_/dt = *J*_6_ − *J*_7_	0	NA

**Flow**		**Biological role**	**Phenomenological coefficients (*k*)**	**References**

*J*_0_ = *k*_0_ × *R* × (1 + *Q*_2__*A*_)		Enter of resources allocated for protein synthesis	3.9E-06	Odum, 1996
*J*_1_ = *k*_1_ × *Q*_1_		Host-cell RNA transcription and translation	6.9E-05	Eden et al., 2011; Kummer et al., 2014
*J*_2__*A*_ = *k*_2__*A*_ × *Q*_1_		Short-half-life protein synthesis	2.6E-05	Adelman et al., 2002; Klumpp and Hwa, 2008; Eden et al., 2011
*J*_2__*B*_ = *k*_2__*B*_ × *Q*_1_ × (1 + *Q*_4_)		Long-half-life protein synthesis	1.6E-05	Proshkin et al., 2010; Eden et al., 2011
*J*_3_ = *k*_3_ × *Q*_1_ × *Q*_2__*B*_ × *Q*_3_ × *Q*_5_		Virus-RNA replication	1.0E-01	Mahmoudabadi et al., 2017
*J*_4_ = *k*_4_ × *Q*_3_		Viral RNA translation	6.9E-05	Kummer et al., 2014
*J*_5_ = *k*_5_ × *Q*_1_ × *Q*_2__*B*_ × *Q*_4_		Recruitment of resources and host-cell protein machinery for virion assembly	1.7E-03	Mahmoudabadi et al., 2017
*J*_6_ = *k*_6_ × *Q*_1_ × *Q*_2__*B*_ × *Q*_4_		Virion assembly	3.0E-03	Mahmoudabadi et al., 2017
*J*_7_ = *k*_7_ × *Q*_1_ × *Q*_2__*B*_ × *Q*_5_		Virion budding	8.3E-04	Mahmoudabadi et al., 2017
*J*_13_ = *k*_13_ × *Q*_2__*B*_ × *Q*_1_ × *Q*_3_ × *Q*_5_		Flow of host-cell resources diverted to let virus enter	8.3E-02	Mahmoudabadi et al., 2017
*J*_15_ = *k*_15_ × *Q*_1_ × *Q*_4_ × *Q*_2__*B*_		Flow of host-cell resources diverted to let virion assembly	1.7E-03	Mahmoudabadi et al., 2017
*J*_17_ = *k*_17_ × *Q*_1_ × *Q*_5_ × *Q*_2__*B*_		Flow of host-cell resources diverted to let virion shedding	8.3E-04	Mahmoudabadi et al., 2017
*J*_20__*A*_ = *k*_20__*A*_ × *Q*_2__*A*_		Flow of host-cell short-half-life proteins addressed to degradation	3.9E-06	Eden et al., 2011; Boisvert et al., 2012
*J*_20__*B*_ = *k*_20__*B*_ × *Q*_2__*B*_		Flow of host-cell long-half-life proteins addressed to degradation	1.6E-06	Eden et al., 2011; Boisvert et al., 2012
*J*_21__*A*_ = *k*_21__*A*_ × *Q*_2__*A*_		Proteostasis mechanisms, including proteasome degradation and autophagy to re-cycle unfolded, old or not functional host-cell short-half-life proteins	3.9E-06	Eden et al., 2011; Boisvert et al., 2012
*J*_21__*B*_ = *k*_21__*B*_ × *Q*_2__*B*_		Proteostasis mechanisms, including proteasome degradation and autophagy of to re-cycle unfolded, old or not functional host-cell long-half-life proteins	3.9E-06	Eden et al., 2011; Boisvert et al., 2012
*J*_23_ = *k*_23_ × *Q*_1_ × *Q*_2__*B*_ × *Q*_3_ × *Q*_5_		Flow of host-cell proteins recruited to let virus enter and RNA transcription	8.3E-02	Mahmoudabadi et al., 2017
*J*_25_ = *k*_25_ × *Q*_1_ × *Q*_2__*B*_ × *Q*_4_		Flow of host-cell proteins recruited to let virion assembly	1.7E-03	Mahmoudabadi et al., 2017
*J*_27_ = *k*_27_ × *Q*_1_ × *Q*_2__*B*_ × *Q*_5_		Flow of host-cell proteins recruited to let virion budding	1.7E-03	Mahmoudabadi et al., 2017
*J*_35_ = *k*_35_ × *Q*_4_		Flow of viral RNA to embed in the virion	4.6E-05	Mahmoudabadi et al., 2017
*J*_50_ = *k*_50_ × *Q*_1_ × *Q*_2__*B*_ × *Q*_5_		Virion shedding	4.6E-05	Mahmoudabadi et al., 2017

* Equations representing the dynamics of the stocks and calibration values are also shown. Calibration value is expressed in ×10^12^ ATP-eq.

**TABLE 2:** Relevant flows included for the diagram and simulator development.

Flow	Description	Value	Stock turnover time	References
*J*_1_	Host protein translation		4 h	Kummer et al., 2014
*J*_1_ + *J*_2__*A*_	Protein synthesis rate	10–20 aa/s		Kudva et al., 2013
*J*_1_ + *J*_2__*B*_	Protein synthesis rate	10–20 aa/s		Kudva et al., 2013
*J*_2__*A*_	Host protein transcription and translation (long-half-life proteins)	13 aa/s		Adelman et al., 2002; Klumpp and Hwa, 2008
*J*_2__*A*_	Host protein transcription and translation (long-half-life proteins)	2–500 (140) mRNA/h 1,000/proteins/mRNA/h 660 mRNA/h/cell	1 h	Howard-Ashby et al., 2006; Ben-Tabou De-Leon and Davidson, 2009; Schwanhausser et al., 2011
*J*_2__*A*_	Host protein transcription and translation (long-half-life proteins)	60,200 mRNA/h to convert in ATP eq		Pelechano et al., 2010
*J*_2__*B*_	Host protein transcription and translation (short half-life proteins)	42 nt/s + 14 aa/s		Proshkin et al., 2010
*J*_2__*A*_ + *J*_2__*B*_		15% 20% respiration rate	0.99 μmol/*k*g/day	Buttgereit and Brand, 1995; Waterlow, 2006
*J*_20_	Protein degradation	0.08/h		Doherty et al., 2009
*J*_20_	Turnover rate of protein	20/hs		Boisvert et al., 2012
*J*_13_	Virus transcription and replication	2,500 nt/s	12 s	Mahmoudabadi et al., 2017
*J*_3_	Virus transcription and replication	2,500 nt/s	12 s	Mahmoudabadi et al., 2017
*J*_4_	Viral protein translation		4 h	Kummer et al., 2014
*J*_5_	Virion assembly		10 min	Mahmoudabadi et al., 2017
*J*_6_	Virion assembly		10 min	Mahmoudabadi et al., 2017
*J*_7_	Virion budding		20 min	Mahmoudabadi et al., 2017
*J*_50_	Virion shedding	24% in 21.6 h	21.6 h	Murphy et al., 1980
*J*_15_	Virion assembly		10 min	Mahmoudabadi et al., 2017
*J*_17_	Virion budding		20 min	Mahmoudabadi et al., 2017
*J*_23_	Virus transcription and replication	2,500 nt/s	12 s	Mahmoudabadi et al., 2017
*J*_25_	Virion assembly			Mahmoudabadi et al., 2017
*J*_27_	Virion budding		20 min	Mahmoudabadi et al., 2017
*J*_21_	Protein recycling of short and long half unfolded-defective proteins	0.08/h		Doherty et al., 2009
*J*_3_ + *J*_4_ + *J*_5_ + *J*_6_ + *J*_7_ + *J*_50_	vRNA replication and virion shedding, virus production after infection		6 h	Sedmak and Grossberg, 1973; Baccam et al., 2006

### Development of the Virus–Host Interaction Systemic Simulator

After setting the initial conditions at time 0 for the stocks, system solutions were obtained using recursive computation for a relative short period of time (identified with the median life of an epithelial cell, 7 days), in order to appreciate the model dynamic behavior. The computational model based on a set of differential equations that describe the rates of change of all stocks in the ST diagram (Odum and Odum, 2000; Bossel, 2007) was developed using the open-source software package SCILAB^1^, which uses approximation techniques to evaluate stocks.

Given a set of initial conditions for the stocks (i.e., the initial state of the system) and a set of phenomenological coefficients *k* associated to flows, the set of interconnected equations was treated by a standard finite-different method, taking care of choosing a time step short enough to evidence the dynamics of any of the studied processes. The coefficients *k*_i_ were calculated, on the basis of literature data, considering the dynamics of each single stock, by quantifying flows and stocks during the time interval set as simulation step, as shown in Table 1. When different flows coparticipate in a process, each coefficient gathers all the actions that concur to the intensity of the outcoming flow(s). In detail, the parameters used to run the model (i.e., the set of values for the *k*_i_ coefficients), describing the reaction of each system component to a change in any other one, were derived from the stocks turnover times (Odum and Odum, 2000; Bossel, 2007). Therefore, the host–virus interaction computational model, built on experimental evidences as listed in Tables 1, 2, is not specific for a unique virus, but may represent the patterns of any virus–host interaction, in which stocks, flows, and processes are those relevant for the operation of the system at issue.

The reliability of both available data and modeling was tested by evaluating the effect of the variation of each of the most relevant input data (stocks and processes) on the system trajectories. Unfortunately, here is not a single comprehensive procedure suitable for the validation of all dynamic models, being dependent on their usefulness, in turn referred to the very purpose of the model itself (Grüne-Yanoff and Weirich, 2010). We chose the sensitivity analysis approach (Qudrat-Ullah, 2012; Hekimoğlu and Barlas, 2016), which allows to see to what extent a variation on these values can lead to alternative evaluations of the system dynamics. In particular, we applied a 50% variation (either positive or negative) to those parameters that the results were more sensitive to. As expected, while the corresponding simulations varied as well, the general patterns presented in the following remained the same, especially concerning the overall trends shown by comparing the groups of simulations, providing a model validation.

## Results

### Stock-Flow Diagram of (+)ssRNA Virus–Host Interaction

**FIGURE 1:**
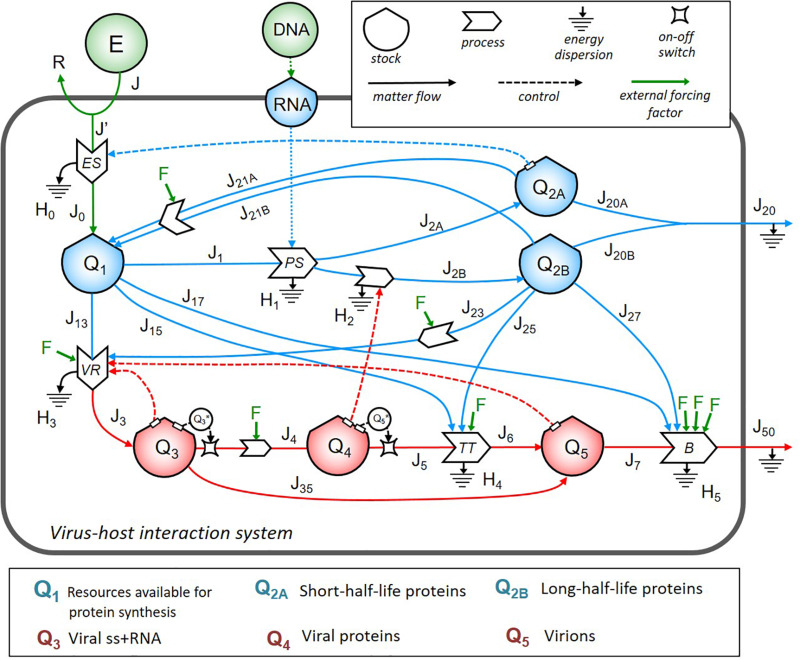
The energy systemic diagram of a cell infected by ss+ RNA virus. Stock-flow diagram of the virus–host interaction system. In the upper right box are the meaning of symbols. The color code is as follows: blue for host cell energy stocks and relative inflows and outflows; red for virus energy stocks and relative inflows and outflows; green for external energy inputs and external driving forces *F* corresponding to different therapeutic strategies. The lower box lists the biological contents of the stocks, all expressed in terms of energy (ATP-equivalent units).

First, we identified the important structures in the system and then used to build up the stock-flow diagram of the virus–host interaction system. In Figure 1, symbols were borrowed from the energy language (Odum and Odum, 2000; Brown, 2004): shields indicate the stocks; big solid arrows indicate the processes; line arrows indicate the flows; dashed lines show the controls exerted by the stocks on the processes.

The dynamics of energy allocation for protein synthesis contained in the stock *Q*_1_ depended on the cell bioenergetics, e.g., the number of mitochondria, OX-PHOS activity levels, and cell cycle phase (Murayama et al., 2008; Canto et al., 2009; Li et al., 2020). In the absence of virus, the stationary configuration was given by energy required to flow from stock *Q*_1_ (via *J*_1_, *J*_2__*A*_, and *J*_2__*B*_ flows) to stocks *Q*_2__*A*_ and *Q*_2__*B*_ to produce, respectively, short- and long-half-life proteins, which could, in turn, be recruited by VR machinery. *J*_20__*A*_ and *J*_20__*B*_, grouped into the flow *J*_20_, represented the outflow of folded, fully functional proteins addressed to secretion or surface exposure. Based on basal proteostasis of host cell, recovery of energetic sources from proteins not addressed to leave the system could be possible via several complex processes (e.g., proteasomal degradation, and autophagy), identified by flows of materials *J*_21__*A*_ and *J*_21__*B*_, respectively, from *Q*_2__*A*_ and *Q*_2__*B*_ back to *Q*_1_.

The viral load in the system, expressed by the stocks *Q*_3_ (identified as viral RNA content to be used for viral transcription and translation), *Q*_4_ (translated viral proteins content), and *Q*_5_ (full assembled virions to shed virus outside), diverted, at the time of infection, resources directly from *Q*_1_ (through flows *J*_13_, *J*_15_, and *J*_17_) and *Q*_2__*B*_ (through flows *J*_23_, *J*_25_, and *J*_27_). Virions shedding was represented by the flows *J*_7_ and *J*_50_ through the contribution of the host flows *J*_17_ and *J*_27_. The output flows *J*_4_ and *J*_5_ were set to be effective only if the value of the respective stock *Q*_3_ and *Q*_5_ was higher than a threshold, as represented by the two switch symbols in the diagram.

We identified four feedback loops (represented by dot lines in Figure 1): (i) the positive control of *Q*_2__*A*_ stock on the energy supply process (occurring when more structural host proteins operate to maintain the energetics homeostasis of the host cell); (ii) the positive control of *Q*_3_ stock on the VR process (highlighting that the more viral RNA is in the system, the more intensive replication can occur if host sources are available); (iii) the positive control of *Q*_4_ stock on the processes of synthesis and maturation of host proteins (highlighting that the more viral proteins are made, the more host proteins are synthesized to be recruited in the virion assembly machinery, increasing *J*_2__*B*_); (iv) the positive control of *Q*_5_ stock on the VR process (highlighting that the more virions are produced, the more resources are diverted from the host cell to viral replication).

### System Dynamics of (+)ssRNA Virus–Host Interaction

**FIGURE 2:**
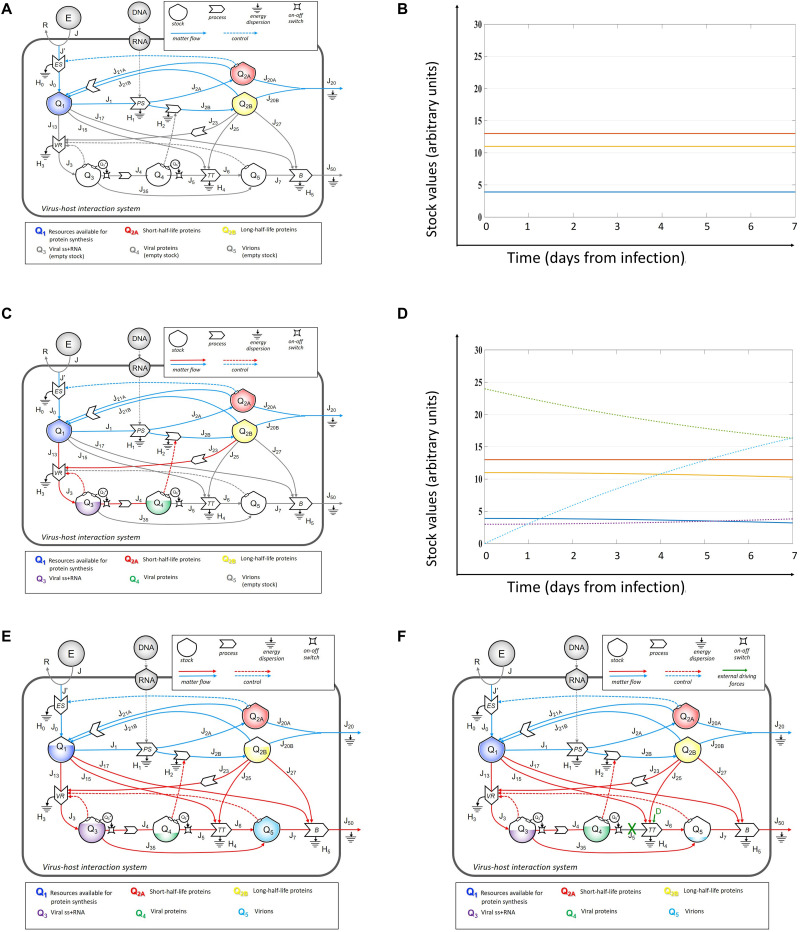
Systems configurations based on initial conditions and effects of external driver forces. In the configuration of initial null viral load **(A)**, the value of stocks *Q*_1_, *Q*_2__*A*_, and *Q*_2__*B*_ were constant, and the system behavior was stationary **(B)**, with constant values of all stocks overtime. At time of infection, the network of flows and feedbacks identified a new configuration **(C)**, to generate a not stationary pattern **(D)**, in which stock values change overtime in response to the other elements of the system, which can evolve to maximize the virions’ stock **(E)**. Application of external driving forces, acting on processes (identified by red cross on *J*_5_), can reduce the flows and address new configurations **(F)**, identifying leverage points that can be explored at different magnitude and timepoints with a computational simulator.

First, a computational model was derived from the stock-flow diagram shown in Figure 1 using the standardized workflow of systemic modeling (Odum and Odum, 2000; Xue et al., 2018). Figure 2 shows two different system self-organized patterns (configurations) to guide reader in the overall comprehension of the proposed approach. The virus–host interaction was represented as an evolving set of simulated trajectories, to which the positive value of *Q*_3_ stock had given access, using preexisting stocks, processes, and flows of the host cell, followed overtime by progressive filling of *Q*_4_ and *Q*_5_ stocks. In (Figure 2A) configuration, the viral load is null (the stocks *Q*_3_, *Q*_4_, and *Q*_5_ are empty), and the values of stocks *Q*_1_, *Q*_2__*A*_, and *Q*_2__*B*_ are constant; thus, the system behavior is stationary (Figure 2B). At time of infection, the *Q*_3_ stock was fed, and its proteins could interact with the host proteome to sustain RNA replication. Based on previous works in the field (Wei et al., 1995; Adelman et al., 2002; Mohler et al., 2005; Regoes et al., 2005; De Boer et al., 2010), we identified a time delay of 2–6 h required to record changes in the *Q*_5_ stock.

Moreover, the value of *Q*_5_ varied over time due to changes that occurred at different timepoints in the stocks *Q*_2__*B*_, *Q*_3_, and *Q*_4_. Thus, the network of flows and feedbacks could identify a new configuration (Figure 2C), to generate a non-stationary behavior (Figure 2D), where the values of *Q*_3_, *Q*_4_, and *Q*_5_ stocks evolved in a non-linear way (Supplementary Figure 1), to maximize the value of virions stock in the configuration (Figure 2E). We define the (Figure 2A) configuration as healthy, the (Figure 2C) configuration as early infection associated to asymptomatic disease, the (Figure 2E) configuration as late infection associated to symptomatic disease, and the (Figure 2F) configuration as symbiotic infection, consequent to any approach derived from the application of external driving forces at any time able to maintain configuration (Figure 2C) without crashing the system. The goal for any curative approach should be to recover the (Figure 2A) configuration when an infection occurs.

**FIGURE 3:**
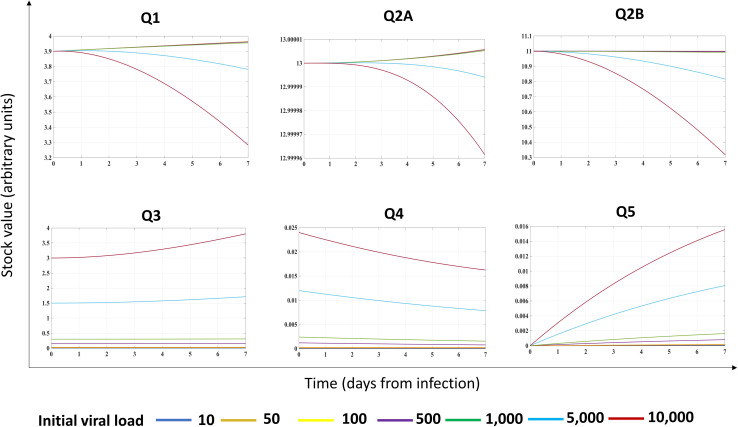
Effects of initial viral load on the energy dynamics of a cell infected by a (+)ssRNA virus. Stock values, expressed in ATP-eq (arbitrary units chosen as proxy), are shown from Day 0 though Day 7 as a function of different initial viral loads (indicated at the bottom with different color codes). Evolutionary pattern for each stock was not linear.

Second, we investigated the system dynamics under different initial conditions, exploring the possible role of different initial viral loads (Figure 3). Assuming different initial viral loads (10–10,000 RNA copies range), we found a threshold (at about 5,000 RNA copies) for triggering the progressive reduction of *Q*_1_ (Figure 3). Indeed, for low initial viral load (10–1,000 RNA copies), the system perturbation could be absorbed by the configuration itself (Supplementary Figure 1), without affecting the overtime stock value of *Q*_1_ and *Q*_2__*B*_ but maintaining constant *Q*_3_, *Q*_4_, and *Q*_5_.

The behavior of stock values *Q*_1_, *Q*_2__*A*_, and *Q*_2__*B*_ diverged non-linearly at the threshold value, with a progressive decrease, starting at Day 3 from infection (cyan and red lines, respectively). We found a non-linear evolution of the system output (the *Q*_5_ stock) depending on the initial conditions: for *Q*_3_ stock in the range (10–1,000 RNA copies), the *Q*_5_ was linear, whereas for higher initial viral load, the growth of *Q*_5_ was linear in the first day and non-linear in the further timeframe, associated to a progressive, unpredictable reduction of *Q*_3_*–Q*_4_ stocks, reflecting in biological terms the turnover of viral proteins required for virions budding.

The non-linear behavior of the (+)ssRNA virus–host interaction was due to the control of *Q*_4_ stock on processes of the host cell, forcing host proteins *Q*_2__*B*_ to favor the production of virions (to feed the *Q*_5_ stock value) through the increased *J*_23_, *J*_25_, and *J*_27_ flows. In biological terms, the progressive *Q*_1_ reduction reflects the metabolic rewiring of infected host cells (Chen et al., 2016), with progressive reduction of resources allocated for the maintenance of host processes, requiring a metabolic shift to less efficient but more rapid source of the available energy required for downstream processes (Thaker et al., 2019). The described system dynamics was experimentally validated for influenza virus (Mahmoudabadi et al., 2017). Results confirmed a previous theoretical assumption, showing a Gibbs free energy for virus lower than its host (Popovic and Minceva, 2020a, b), when virus and host cells are evaluated separately and not as a unique system as herein proposed.

The behavior of *Q*_1_ following different initial values of *Q*_3_ could explain both the variable incubation time in each individual subject and why infections due to (+)ssRNA viruses can occur asymptomatically in most cases. The resilience of virus–host system for a specific range of *Q*_3_ amount could, in turn, depend on intrinsic and extrinsic factors. Indeed, in response to manipulation of stocks and flows, and based on timeframe of observation, the system could evolve along different, non-linear trajectories, requiring early intervention upon infection to make the system resilient to growth of viral stocks.

### System Dynamics of (+)ssRNA Virus–Host Interaction in Response to External Driving Forces Applied to Reduce Virions Outflow

Currently, the search for a therapeutic strategy is based on single target-related parameters, while we propose to identify systemic targets (i.e., polytarget) to improve host response to host–virus interaction. Starting from the pharmacodynamics of compounds currently under investigation for a typical (+)ssRNA virus (Thorlund et al., 2020), we could reclassify them, based on their systemic mechanisms of action as listed in Table 3. Their effects may be potentially simulated to establish the single-cell effect, the best time, and/or schedule of administration, as shortcut of *in vitro* studies, with a detail level established on the basis of the purpose of the study design. To this end, we applied the search of systemic leverage points by simulating the dynamics of multiple scenarios, upon the action of a generic external driving force (*D*), assuming that the minimization of the value of *Q*_5_ stock over time should limit the propagation of virions outside the cells.

**TABLE 3:** Examples of drugs that could act as external forcing factors on identified systemic flow targets.

Flow target(s)	Compound	Mechanism of action	References
*J*_0_, *J*_3_, *J*_4_, *J*_21__*A*_, *J*_21__*B*_	FK506 (tacrolimus)	FKBP15 inhibitor. ER protein quality control regulators. Bioenergetics regulators. mRNA translation inhibitor.	Wiederrecht et al., 1993; Gordon et al., 2020b
*J*_0_, *J*_4_, *J*_21__*A*_, *J*_21__*B*_	Rapamycin		Jefferies et al., 1997; Wang et al., 2014; Gordon et al., 2020b
*J*_1_, *J*_3_, *J*_21__*B*_	Valproic acid	HDAC2 inhibitor	Cong and Bacchetti, 2000; Phiel et al., 2001; Nagesh et al., 2017; Gordon et al., 2020b
*J*_1_, *J*_4_	SAHA	pan HDAC inhibitors	Saha and Parks, 2019
*J*_1_, *J*_35_, *J*_2__*B*_	Selinexor	mRNA nuclear export complex inhibitor	Bardina et al., 2009; Castelló et al., 2009; Schmidt et al., 2013; Walker et al., 2013; Watters et al., 2017; Gordon et al., 2020b
*J*_4_, *J*_2__*A*_, *J*_2__*B*_	Dabrafenib	Kinase inhibitor, protein synthesis inhibitor	Burkard et al., 2015; Kindrachuk et al., 2015; Gordon et al., 2020b
*J*_0_, *J*_13_	Metformin	Inhibitor of respiratory electron transport, glycolysis regulation	Fontaine et al., 2015; Gordon et al., 2020b
*J*_3_, *J*_13_	Camostat, nafamostat	TMPRSS inhibitors proteolytic cleavage of viral spi*k*e protein priming to the receptor ACE2 present in human cell	Gordon et al., 2020b; Kailas et al., 2020
*J*_4_, *J*_2__*A*_, *J*_2__*B*_	Ponatinib	Kinase inhibitor, protein synthesis inhibitor	Burkard et al., 2015; Gordon et al., 2020b
*J*_3_	Ribavirin	Nucleoside inhibitor (mutagenic ribonucleoside)	Patterson and Fernandez-Larsson, 1990; Crotty et al., 2000; Gordon et al., 2020b
*J*_13_	Chloramphenicol, tigecycline, linezolid	Antibiotics, able to inhibit mitochondrial ribosomes	Colberg-Poley et al., 2000; Gordon et al., 2020b
*J*_21__*A*_, *J*_21__*B*_	Chloroquine	SIGMAR1/SIGMAR2 inhibitor. Autophagy inhibitor.	Keyaerts et al., 2004; Vincent et al., 2005; Saeed et al., 2011; Cottam et al., 2014; Borba et al., 2020; Gordon et al., 2020b
*J*_3_, *J*_5_, *J*_21__*A*_, *J*_21__*B*_	Hydroxychloroquine	Autophagy inhibitor, antiviral effect	Cottam et al., 2014; Gautret et al., 2020; Gordon et al., 2020b; Liu et al., 2020
*J*_1_, *J*_4_, *J*_5_	Zotatifin, ternatin 4, tomvosertib	mRNA translation inhibitors	Gordon et al., 2020b
*J*_1_, *J*_2__*B*_, *J*_23_	Silmitasertib or TMCB	Casein *k*inase II inhibitors, protein synthesis inhibitor	Siddiqui-*J*ain et al., 2010; Reineke et al., 2017; Gordon et al., 2020b
*J*_3_	Remdesivir	Nucleoside analog, interferes with RNA-dependent RNA polymerase	Sheahan et al., 2017
*J*_13_	Umifenovir	Inhibitor of the fusion between the viral envelope (surrounding the viral capsid) and the cell membrane of the target cell	Shi et al., 2007; Pécheur et al., 2016
*J*_13_	Lisinopril, losartan	ACE inhibitors, prevent the fusion between the viral envelope and the cell membrane of the target cell	Hoffmann et al., 2020
*J*_3_	Favipiravir	Selective inhibitor of RNA-dependent RNA polymerase	Cai et al., 2020; Shiraki and Daikoku, 2020
*J*_2__*A*_, *J*_2__*B*_, *J*_3_*, *J*_4_, *J*_7_*, *J*_21_	Macrolide antibiotics azithromycin*, clarithromycin	Inhibition of ribosomal translation Autophagy inhibition	Tenson et al., 2003; Stamatiou et al., 2009; Gielen et al., 2010; Petroni et al., 2020

*^∗^Close to Js, refers to azithromycin, in the other colum, being the compount impactinng on the identified flows.*

**FIGURE 4:**
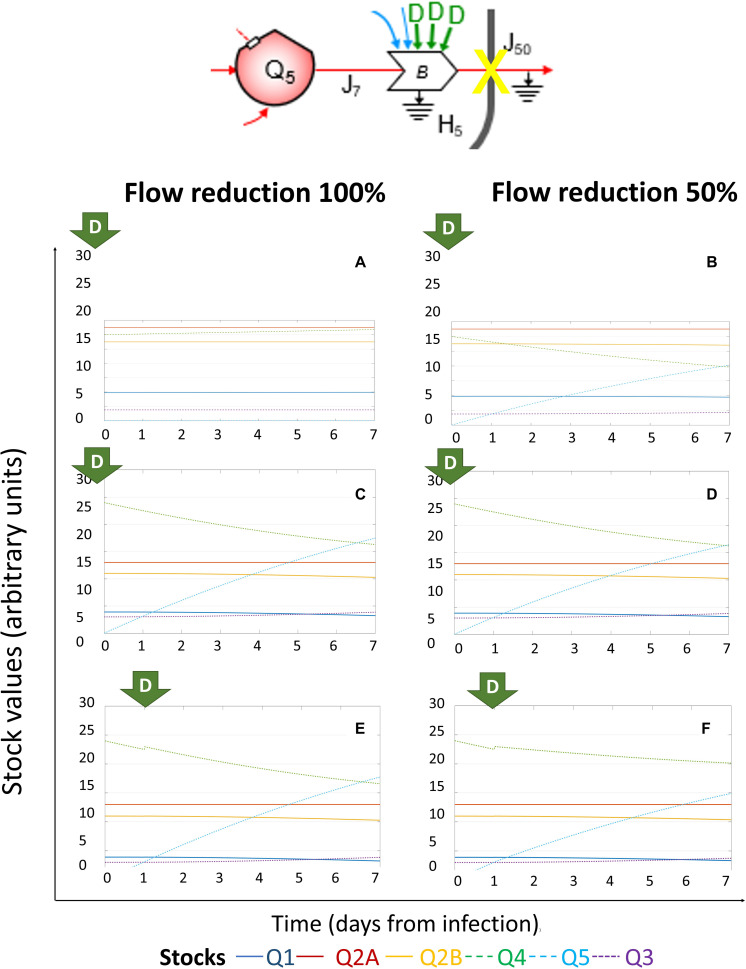
System dynamics of (+)ssRNA virus–host interaction in response to external driving forces applied to reduce virions outflow. Changes over time of the values of each stock of the system diagrammed in Figure 1 (for the color code, see bottom), expressed in ATP-eq, in response to reduction of *J*_50_ (flow of energy required for virions budding). Several scenarios are shown: initial viral load 5k and application of full (100%, **A**) or partial (50%, **B**) *J*_50_ reduction at Day 0; initial viral load 10k and application of full (100%, **C**) or partial (50%, **D**) *J*_50_ reduction at Day 0; initial viral load 10k and application of full (100%, **E**) or partial (50%, **F**) *J*_50_ reduction at Day 1.

First, we explored the system configurations upon reduction of the outflows from the *Q*_5_ virions stock, via manipulation of *J*_7_ and/or *J*_50_. However, minimization of *J*_7_ was counter-effective, due to the increase of *Q*_5_ as consequence of the feedback action in the VR process (data not shown). The effects of full (100%) or partial (50%) reduction of *J*_50_ (flow of energy required for virions budding) could prevent the outflow from *Q*_5_ without stopping its growth, diverting resources from *Q*_1_ and *Q*_2__*B*_ to *Q*_5_, so further supporting viral hijacking of cellular metabolism and impairing host cell homeostasis. As shown in Figure 4, the effect of driving forces acting to modulate *J*_50_ was different based on application time, Day 0 (Figures 4A–D) versus Day 1 (Figures 4E,F), and initial viral load, as the early abrogation of *J*_50_ when the amount of *Q*_3_ was 5,000 RNA copies could restore the stationary status (Figure 4A), while halving *J*_50_ maintained homeostasis for host-cell stock, but could not prevent the growth of *Q*_5_ (Figure 4B). At increasing initial viral load, reduction of *J*_50_ applied at Day 0 (Figures 4C,D) or at Day 1 (Figures 4E,F) could not prevent the growth of *Q*_5_ and the progressive decrease of *Q*_1_ and *Q*_2__*B.*_ This systemic dynamics can explain the relationship between the time of initiation of neuraminidase inhibitors and their efficacy (Moscona, 2005), as treatment starting within the first 12 h after the onset of fever shortened the illness by more than 3 days, as compared with treatment starting at 48 h (Aoki et al., 2003).

### System Dynamics of (+)ssRNA Virus–Host Interaction in Response to External Driving Forces Applied to Reduce Virions Assembly

Second, we explored the systemic response to full (100%) or partial (50%) reduction of either *J*_3_ (flow of energy required for RNA replication), or *J*_4_ (flow of energy required for viral RNA translation and viral protein synthesis), or *J*_5_ (flow of energy required for virions assembly), which are involved in virions assembly and are typically dependent on intrinsic viral biological properties.

**FIGURE 5:**
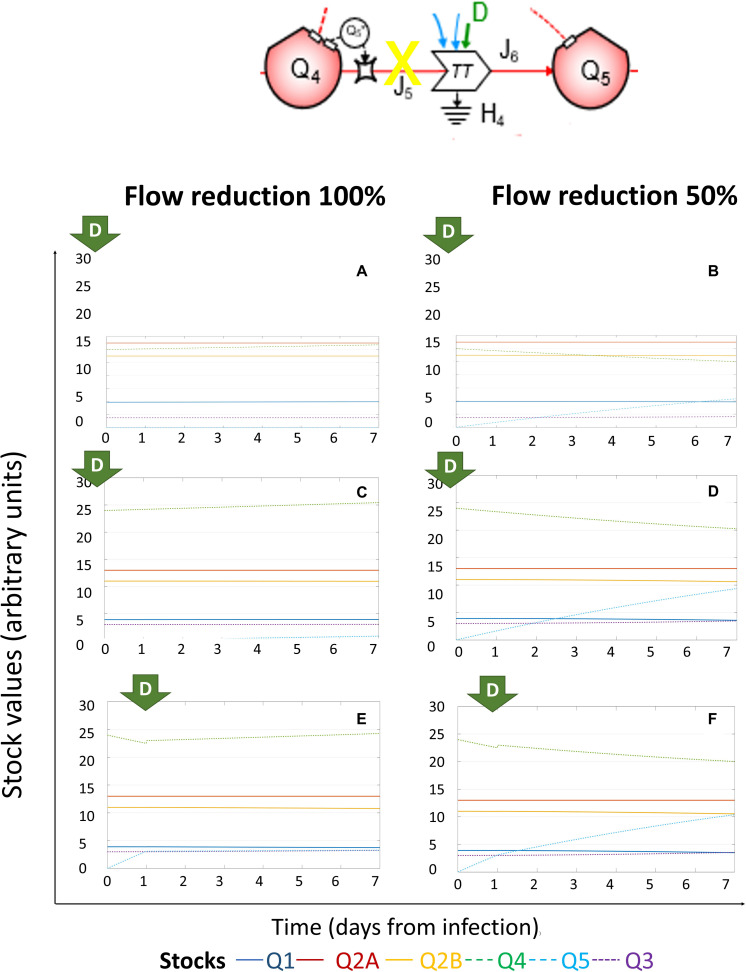
System dynamics of (+)ssRNA virus–host interaction in response to external driving forces applied to reduce virions assembly. Changes over time of the values of each stock of the system diagrammed in Figure 1 (for the color code, see bottom), expressed in ATP-eq, in response to reduction of *J*_5_ (flow of energy required for virions assembly). Several scenarios are shown: initial viral load 5k and application of full (100%, **A**) or partial (50%, **B**) *J*_5_ reduction at Day 0; initial viral load 10k and application of full (100%, **C**) or partial (50%, **D**) *J*_5_ reduction at Day 0; initial viral load 10k and application of full (100%, **E**) or partial (50%, **F**) *J*_5_ reduction at Day 1.

Full reduction of *J*_5_ at Day 0 could recover the systems dynamics in a stationary status, in all scenarios of initial viral load tested (5,000 RNA copies, Figure 5A; 10,000 RNA copies, Figure 5C), with constant values for all stocks. A partial reduction of *J*_5_ at Day 0 maintained a stationary status only for lower initial viral load (5,000 RNA copies, Figure 5B) associated to a reduced amount of stocks *Q*_4_ and *Q*_5_ (Figure 5D). The abrogation of *J*_5_ at Day 0 was still effective to preserve the stationary status (Figure 5E), but halving *J*_5_ at Day 1 could reduce but not prevent the growth of *Q*_5_ (Figure 5F). We also simulated the effect of applying the same external inputs at different times: after 1 (Supplementary Figure 2), 3 (Supplementary Figure 3), or 5 days (Supplementary Figure 4) from the initial infection, confirming the role of early application of external driving forces to recover the stationary status of the system.

The importance of full abrogation of *J*_5_ flow for a (+)ssRNA virus–host interaction has been indirectly confirmed by the data recently published by Gordon et al., who cloned, tagged, and expressed 26 of 29 severe acute respiratory syndrome (SARS)–CoV-2 proteins individually in HEK293T cells and used mass spectrometry to measure protein–protein interactions (Gordon et al., 2020b), to identify 69 existing drugs, known to target host proteins or associated pathways, which interact with SARS-CoV-2, addressing the importance to target the host–virus interaction at the level of RNA translation.

### System Dynamics of (+)ssRNA Virus–Host Interaction in Response to External Driving Forces Applied to Reduce Viral Protein Synthesis

**FIGURE 6:**
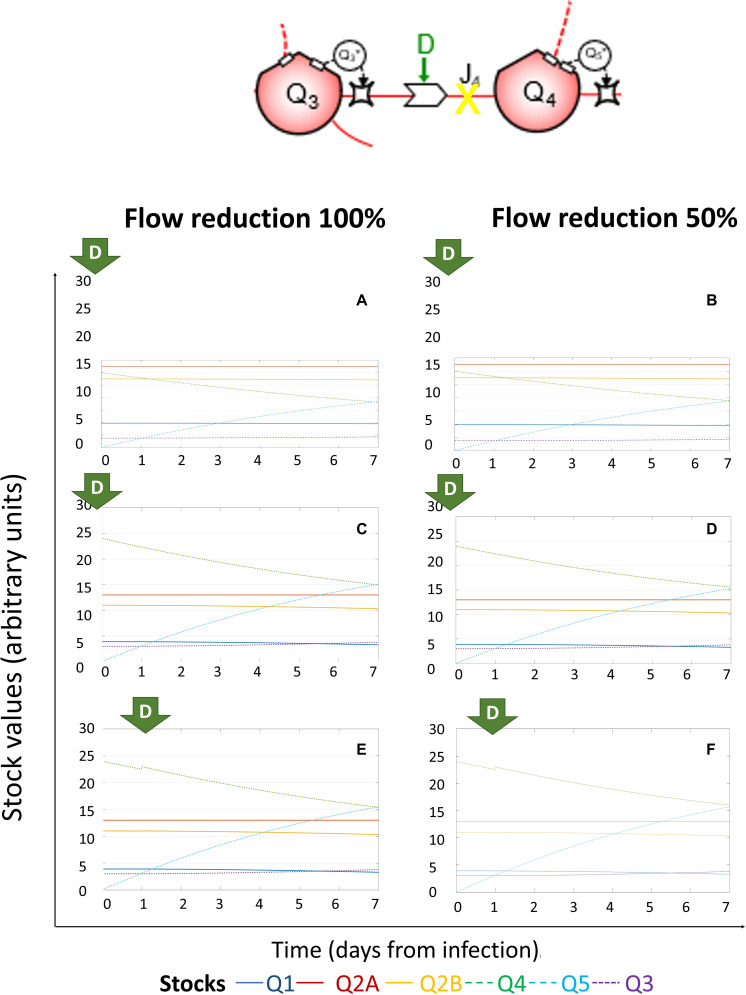
System dynamics of (+)ssRNA virus–host interaction in response to external driving forces applied to reduce viral protein synthesis. Changes over time of the values of each stock of the system diagrammed in Figure 1 (for the color code, see bottom), expressed in ATP-eq, in response to reduction of *J*_4_ (flow of energy required for viral RNA translation and viral protein synthesis). Several scenarios are shown: initial viral load 5k and application of full (100%, **A**) or partial (50%, **B**) *J*_4_ reduction at Day 0; initial viral load 10k and application of full (100%, **C**) or partial (50%, **D**) *J*_4_ reduction at Day 0; initial viral load 10k and application of full (100%, **E**) or partial (50%, **F**) *J*_4_ reduction at Day 1.

Full (100%) or partial (50%) reduction of *J*_4_ (flow of energy required for viral RNA translation and viral protein synthesis) at Day 0 did not affect the dynamics of the system (Figure 6). In particular, for lower initial viral load (5,000 RNA copies, Figures 6A,B), the *Q*_5_ stock was always lower than *Q*_2__*A*_, thus not affecting *Q*_1_, which remained constant. However, when *Q*_5_ stock was greater than *Q*_2__*A*_, *Q*_1_ started to decrease, again suggesting that the effect of viral infection on host metabolism is associated to threshold values specific for each system, and not for each cell type. The observation that—based on initial viral load—the higher value of *Q*_5_ is lower than the stable quantity of *Q*_2__*A*_ and *Q*_2__*B*_ for 5k ATP-eq and higher than *Q*_2__*A*_ and *Q*_2__*B*_ for 10k ATP-eq can explain the contribution of initial viral load and configuration of host-cell stocks in the viral fitness, which depends on host cell cycle stage, addressed as initial value of *Q*_2__*A*_ and *K*_2__*A*_ (Wargo and Kurath, 2012).

### System Dynamics of (+)ssRNA Virus–Host Interaction in Response to External Driving Forces Applied to Reduce Viral RNA Replication

**FIGURE 7:**
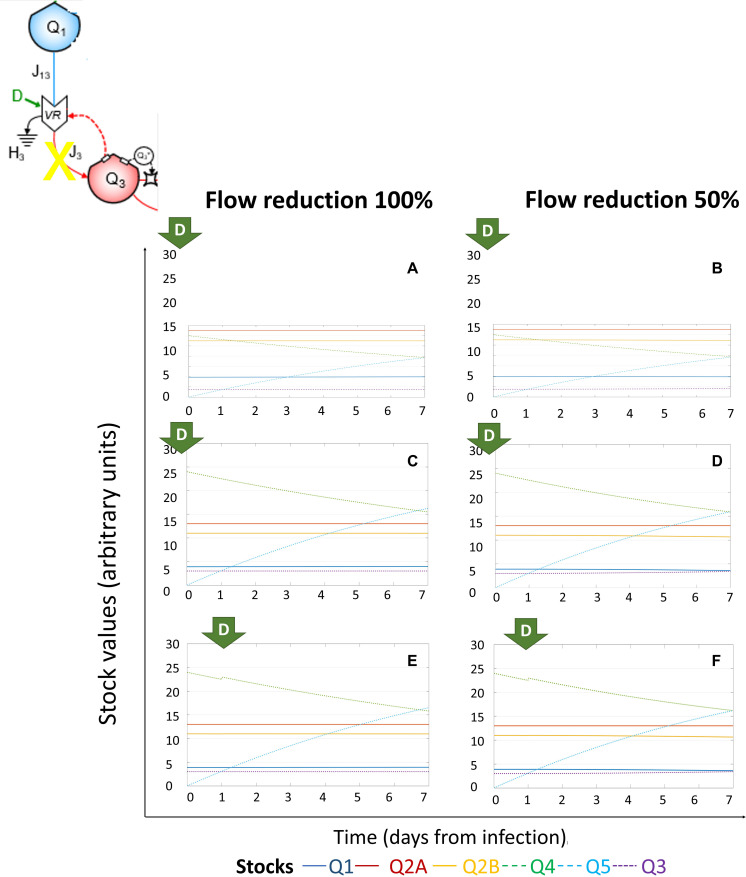
System dynamics of (+)ssRNA virus–host interaction in response to external driving forces applied to reduce viral RNA replication. Changes over time of the values of each stock of the system diagrammed in Figure 1 (for the color code, see bottom), expressed in ATP-eq, in response to reduction of *J*_3_ (flow of energy required for RNA replication). Several scenarios are shown: initial viral load 5k and application of full (100%, **A**) or partial (50%, **B**) *J*_3_ reduction at Day 0; initial viral load 10k and application of full (100%, **C**) or partial (50%, **D**) *J*_3_ reduction at Day 0; initial viral load 10k and application of full (100%, **E**) or partial (50%, **F**) *J*_3_ reduction at Day 1.

Full (100%) or partial (50%) reduction of *J*_3_ (flow of energy required for RNA replication) at Day 0 did not affect the dynamics of the system (Figure 7), but—differently from the previous scenario—*Q*_1_ remained constant even if *Q*_5_ was lower than the stable quantity of *Q*_2__*A*_ and *Q*_2__*B*_, even if administered later after infection (on Day 3 or 5, as shown in Supplementary Figures 2–4). This suggests that the host cell can preserve its homeostasis upon early exposure to inhibitors of viral replication. Interestingly, the pattern in response to single external driving forces was maintained over time, with the values of each stock just shifted, based on the time of application.

Several strategies can be used to reduce selectively virus RNA, including nucleoside analogs, which are metabolized intracellularly into their active ribonucleoside 5′-triphosphate forms and incorporated into the nascent viral RNA by error-prone viral RNA-dependent RNA polymerase (RdRps), to disrupt RNA synthesis directly via chain termination, or accumulation of deleterious mutations in the viral genome. The response to nucleoside analogs could be better tested by our computational model, adding the mutations rate and the DNA/RNA metabolism of host cell, not included in the diagram for lack of experimental data about the turnover of RNA stock, specific for each host cell type of interest.

### System Configurations Dynamics in Response to Multiple External Driving Forces Applied at Different Timepoints From Infection

**FIGURE 8:**
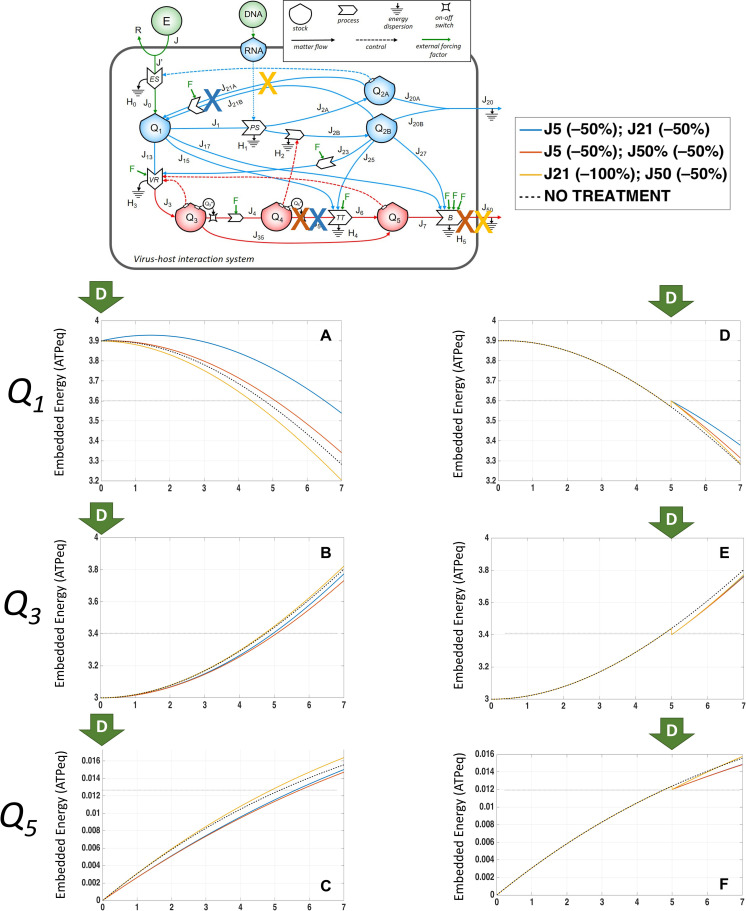
System configurations dynamics in response to multiple external driving forces applied at different timepoints from infection. Changes over time of *Q*_1_ stocks, expressed in ATP-eq, in response to partial (50%) reduction of *J*_5_ and *J*_21_ (blue line), *J*_5_ and *J*_50_ (orange line), *J*_21_ and *J*_50_ (yellow line), or no application of external driving forces applied at Day 0 **(A)** or at Day 5 **(D)** of infection. Changes over time of *Q*_3_ stocks, expressed in ATP-eq, in response to partial (50%) reduction of *J*_5_ and *J*_21_ (blue line), *J*_5_ and *J*_50_ (orange line), *J*_21_ and *J*_50_ (yellow line), or no application of external driving forces applied at Day 0 **(B)** or at Day 5 **(E)** of infection. Changes over time of *Q*_5_ stocks, expressed in ATP-eq, in response to partial (50%) reduction of *J*_5_ and *J*_21_ (blue line), *J*_5_ and *J*_50_ (orange line), *J*_21_ and *J*_50_ (yellow line), or no application of external driving forces applied at Day 0 **(C)** or at Day 5 **(F)** of infection.

Third, we next explored the application of multiple external forcing factors, addressing the requirement of a polytarget approach (Bizzarri et al., 2020). The positive effects on *Q*_1_, *Q*_3_, and *Q*_5_ arising from targeting *J*_5_ was mitigated by the combination with reduction of *J*_50_ or *J*_21_, since the combination targeting *J*_5_ and *J*_21_ (indicated by the blue line in Figure 8) applied at Day 0 could preserve the *Q*_1_ amount better than no treatment (Figure 8A) associated to slower increase of the growth of *Q*_3_ (Figure 8B) and *Q*_5_ (Figure 8C), leading us to explore further combinations. Application of external driving forces acting on *J*_50_ and *J*_21_ (yellow line in Figure 8) increased the *Q*_5_ stock value instead of the expected decrease (Figure 8C); thus, action on a single flow was more efficient than on two of them, given the emergence of compensatory feedbacks and flows. These observations are supported by the controversial findings about the efficacy of macrolides, chloroquine, and their derivatives in the recent COVID-19 pandemic, as their systemic effects include reduction of *J*_5_ and *J*_21_ (Table 3), and could be affected by the viral load and time of application, with weak changes in virions’ spread if applied later in the clinical course of disease as shown by preliminary results of randomized trials (Derwand et al., 2020; Kashour et al., 2020; Magagnoli et al., 2020; Fiolet et al., 2021).

When the combination targeting *J*_5_ and *J*_50_ was applied later (at Day 5 from infection, orange line in Figure 8), *Q*_1_ was higher than untreated (Figure 8D), associated to slower growth of *Q*_3_ (Figure 8E) and *Q*_5_ (Figure 8F), but not leading to restore of stationarity. This behavior is a typical systemic feature, where an intervention on a specific local process may lead to counterintuitive rearrangements in the system dynamics. Thus, once the main flow to reduce was found to identify the *n*-ple stock associated with the desired system output, molecular insights should suggest the biological process to assess a tailored treatment.

## Discussion

In this work, we approached the host–virus interaction dynamics as a systemic problem, and for the first time in the field, we used combined ST tools as a conceptual framework to build up a systemic description of the viral action and host response, critically depending on the existing metabolic environment.

The basic ST idea is to integrate the traditional bottom-up approach—which describes “local” behaviors through cause–effect chains and functional units—with a top-down approach, which points out at the global behavior of the system in terms of its operational configurations, emerging from the feedback-driven response to different driving forces, like those represented, for example, by the chemistry of new drugs (Odum, 1996). The utility of computational simulations stems from their capacity to identify structural side effects, non-linearities, and time delays, which are left unexplained by other approaches. Systems biology already recognized the relevance of complexity in the study of microbiological systems (Kaneko, 2006; Loscalzo and Barabasi, 2011), but although successfully applied in several fields ranging from hard sciences to ecology and economics (Brown, 2004; Brown and Ulgiati, 2004), the potential of ST in the study of biological systems is still underexploited. Thanks to its abstract nature, stock-flow description can be used in a wide range of different fields, realizing the conceptual bridge that connects the language of biological systems to that of ecology.

In targeting the virus–host interaction, there is an emerging need of tools that could early identify those compounds, not primarily designed for their antiviral action, identifiable by *in silico* approaches (Gordon et al., 2020b), which alone or in combination can provide clinical efficacy (Mina et al., 2016; Cheng et al., 2018; Bogdanow et al., 2019; Panja et al., 2019; Gordon et al., 2020b). There are known advantages of *in silico* modeling of the action of therapeutic agents on known diseases through agent-based modeling (Mao et al., 2018). However, the literature evidenced some intrinsic limitations on the choice of parameters, such as the size of investigated populations (Mina et al., 2016), while major problems are related to model validation (Mina et al., 2016; Donkin et al., 2017), also requiring to supplement the models with adequate formal ontologies (Kalfoglou and Schorlemmer, 2003; Gotts et al., 2019).

The proposed model was developed at the single-cell scale. However, in order to define an overall therapeutic approach, the integration with a multiscale approach would be also desirable. In particular, depending on the availability of appropriate data, a future model could focus on different scales, with a more detailed description of some components at the subcellular level, which were grouped (e.g., short- and long-half-life proteins, lipids, and vesicles trafficking) in the present study. On the other hand, the interaction between different cell populations in the host could be also developed, to represent the interaction between healthy and infected cells, and the contribution of immune system (Tan et al., 2007; Katze et al., 2008; Wu et al., 2020) or the repertoire of receptors on the surface of the host cell (Gordon et al., 2020a; Hoffmann et al., 2020; Kailas et al., 2020) to surveil and limit the size of *Q*_3_ stock at the single-cell level. Other natural system constraints could be also included, like some physiological parameters (e.g., temperature and metabolic rate), whose impact on the human body energy dynamics is already understood. The use of a multiscale hierarchical perspective is in principle already possible, as discussed in previous works adopting the same sort of system representation (Ulgiati and Brown, 2009; Ulgiati et al., 2011).

## Conclusion

This work highlights the advantages of applying an ST-based approach to the study of virus–host interaction, being reflected in the possibility of extracting systemic dynamic features that would be otherwise counterintuitive. While a traditional single-target approach would address strategies targeting the viral RNA (*Q*_3_) or the replication process (*J*_3_), our results suggest that the virus growth is more vulnerable if the process of virion growth before expulsion (process TT, involving flow *J*_5_) is targeted.

## Data Availability Statement

The raw data supporting the conclusions of this article will be made available by the authors, without undue reservation, to any qualified researcher.

## Author Contributions

AR designed the study and collected medical knowledge. FG built up the diagram. MC built up the simulator. All authors performed simulation, analyzed data, prepared the figures, and wrote the manuscript.

## Conflict of Interest

The authors declare that the research was conducted in the absence of any commercial or financial relationships that could be construed as a potential conflict of interest.

## Footnotes

1 https://www.scilab.org

## Supplementary Material

Supplementary Figure 1 System dynamics of (+)ssRNA virus–host interaction in response to initial viral load. Changes overtime of the values of each stock of the system diagrammed in Figure 1 (for the color code see at the bottom), expressed in ATP-eq: in absence of infection the system status was stationary **(A)**. Upon infection initial values of *Q*_3_ stock identify the response of system, at 50 **(B)**, 100 **(C)**, 1,000 **(D)**, 5,000 **(E)** and 10,000 **(F)** RNA copies, expressed in ATP-eq. For Q3 stock in the range (10–1,000 RNA copies) the trajectory of *Q*_5_ evolution was linear, while for higher initial viral load the growth of *Q*_5_ was linear in the first day and non-linear in the further timeframe.

Supplementary Figure 2 Effects of targeting leverage point applying external driving force (*D*) at Day 1. Time change of the stocks values (for the color code see at the bottom), expressed in ATP-eq, were depicted over-time, from infection (Day 0, Time 0) through the next 7 days, upon application, at Day 1, of generic external driving forces (*D*) able to reduce the target flows (schemed on the left) of 100% or 50%, respectively.

Supplementary Figure 3 Effects of targeting leverage point applying external driving force (*D*) at Day 3. Time change of the stocks values (for the color code see at the bottom), expressed in ATP-eq, were depicted over-time, from infection (Day 0, Time 0) through the next 7 days, upon application, at Day 3, of generic external driving forces (*D*) able to reduce the target flows (schemed on the left) of 100% or 50%, respectively.

Supplementary Figure 4 Effects of targeting leverage point applying external driving force (*D*) at Day 5. Time change of the stocks values (for the color code see at the bottom), expressed in ATP-eq, were depicted over-time, from infection (Day 0, Time 0) through the next 7 days, upon application, at Day 5, of generic external driving forces (*D*) able to reduce the target flows (schemed on the left) of 100% or 50%, respectively.
