# Supplementary material for: Addressing Non-linear System Dynamics of Single-Strand RNA Virus–Host Interaction
Source: Front Microbiol. 2021 Jan 15;11:600254. doi: 10.3389/fmicb.2020.600254 (PMC7843927; doi:10.3389/fmicb.2020.600254)
Supplement: Supplementary Figure 1 — System dynamics of (+)ssRNA virus–host interaction in response to initial viral load. Changes overtime of the values of each stock of the system diagrammed in Figure 1 (for the color code see at the bottom), expressed in ATP-eq: in absence of infection the system status was stationary (A). Upon infection initial values of Q3 stock identify the response of system, at 50 (B), 100 (C), 1,000 (D), 5,000 (E) and 10,000 (F) RNA copies, expressed in ATP-eq. For Q3 stock in the range (10–1,000 RNA copies) the trajectory of Q5 evolution was linear, while for higher initial viral load the growth of Q5 was linear in the first day and non-linear in the further timeframe. [file Data_Sheet_1.PDF]

Supplementary Material

Addressing dynamic trajectories and leverage points in the virus-host co-evolution: a systems-thinking based approach

Alessandra Romano[1,2], Marco Casazza[2*], Francesco Gonella[3]

Supplementary Figure 1. Early and late changes in stocks values based on viral load at Day 0

Time change of the stocks values, expressed in ATP-eq, from infection (Day 0, Time 0) through the next 7 days, using different initial values in Q_3 (amount of 1,000-5,000-10,000 virions, expressed in ATP-eq). The difference between the value at Day 0, Time 0 and that after 48 hours (at the end of Day 1) or 144 hours (at the end of Day 5) is plotted for each stock.

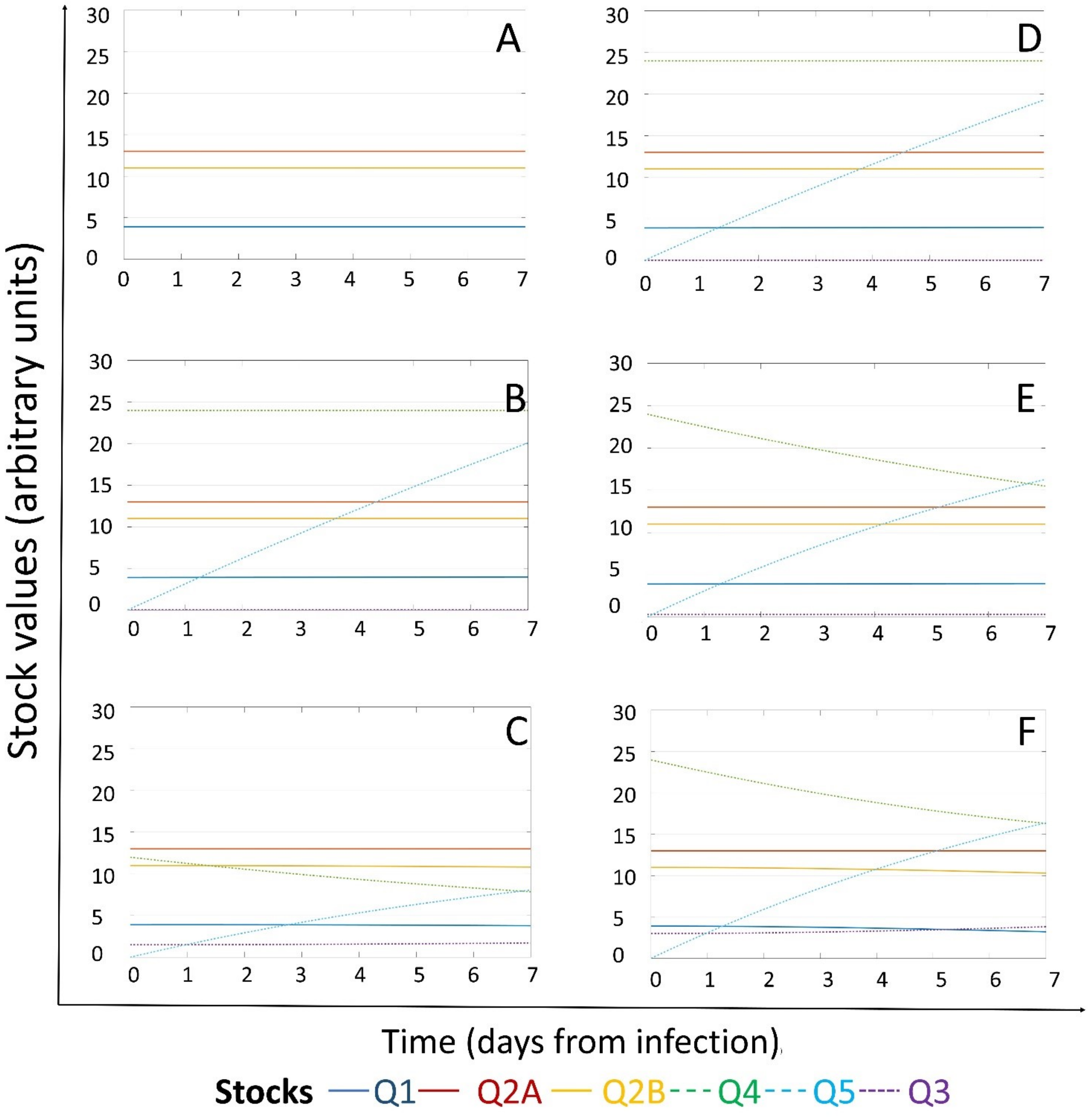

Supplementary Figure 2. Effects of targeting leverage point applying external driving force (*D*) at Day 1.

Time change of the stocks values (for the color code see at the bottom), expressed in ATP-eq, were depicted over-time, from infection (Day 0, Time 0) through the next 7 days, upon application, at Day 1, of generic external driving forces (*D*) able to reduce the target flows (schemed on the left) of 100% or 50%, respectively.

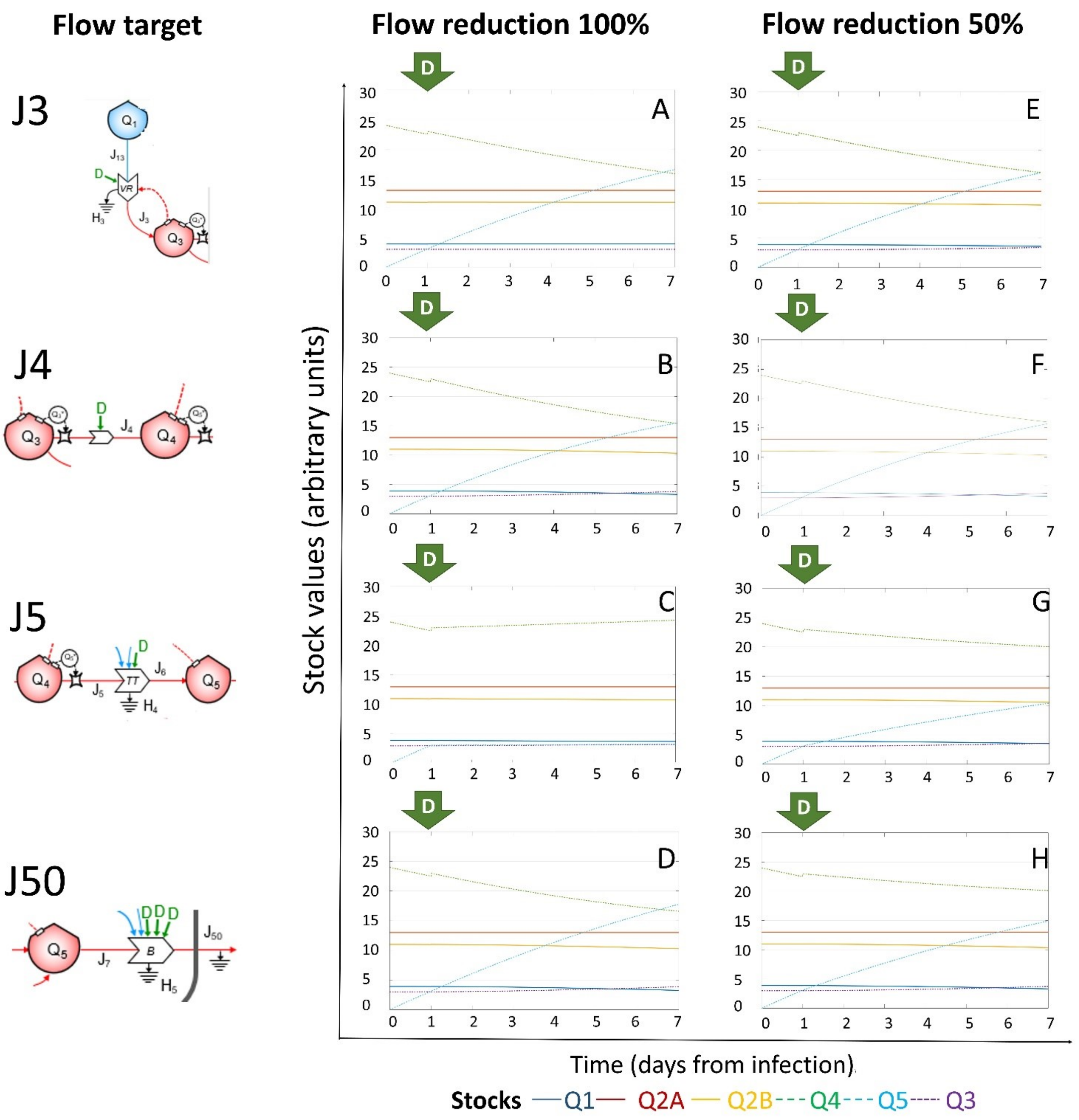

Supplementary Figure 3. Effects of targeting leverage point applying external driving force (*D*) at Day 3.

Time change of the stocks values (for the color code see at the bottom), expressed in ATP-eq, were depicted over-time, from infection (Day 0, Time 0) through the next 7 days, upon application, at Day 3, of generic external driving forces (*D*) able to reduce the target flows (schemed on the left) of 100% or 50%, respectively.

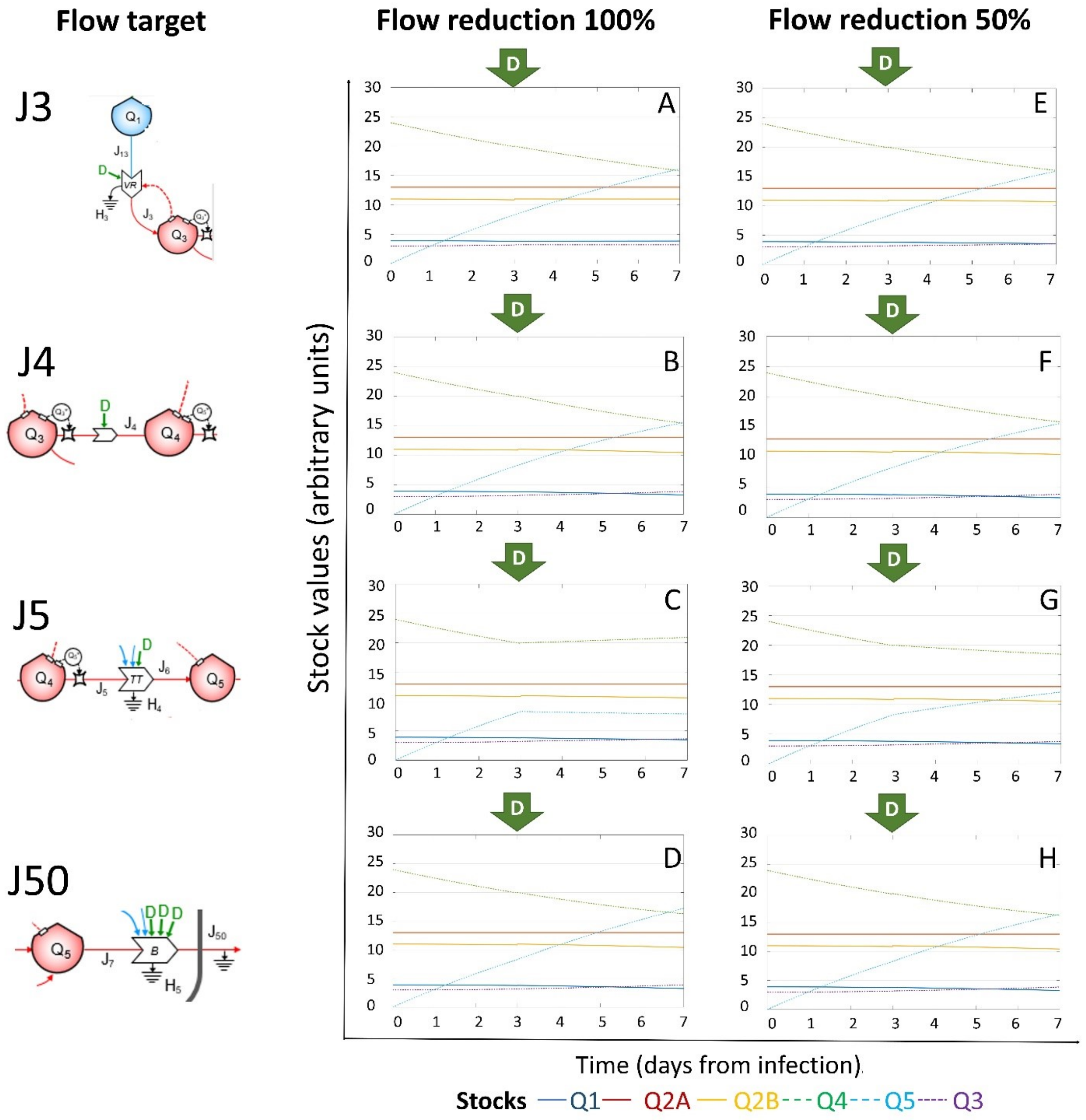

Supplementary Figure 4. Effects of targeting leverage point applying external driving force (*D*) at Day 5.

Time change of the stocks values (for the color code see at the bottom), expressed in ATP-eq, were depicted over-time, from infection (Day 0, Time 0) through the next 7 days, upon application, at Day 5, of generic external driving forces (*D*) able to reduce the target flows (schemed on the left) of 100% or 50%, respectively.

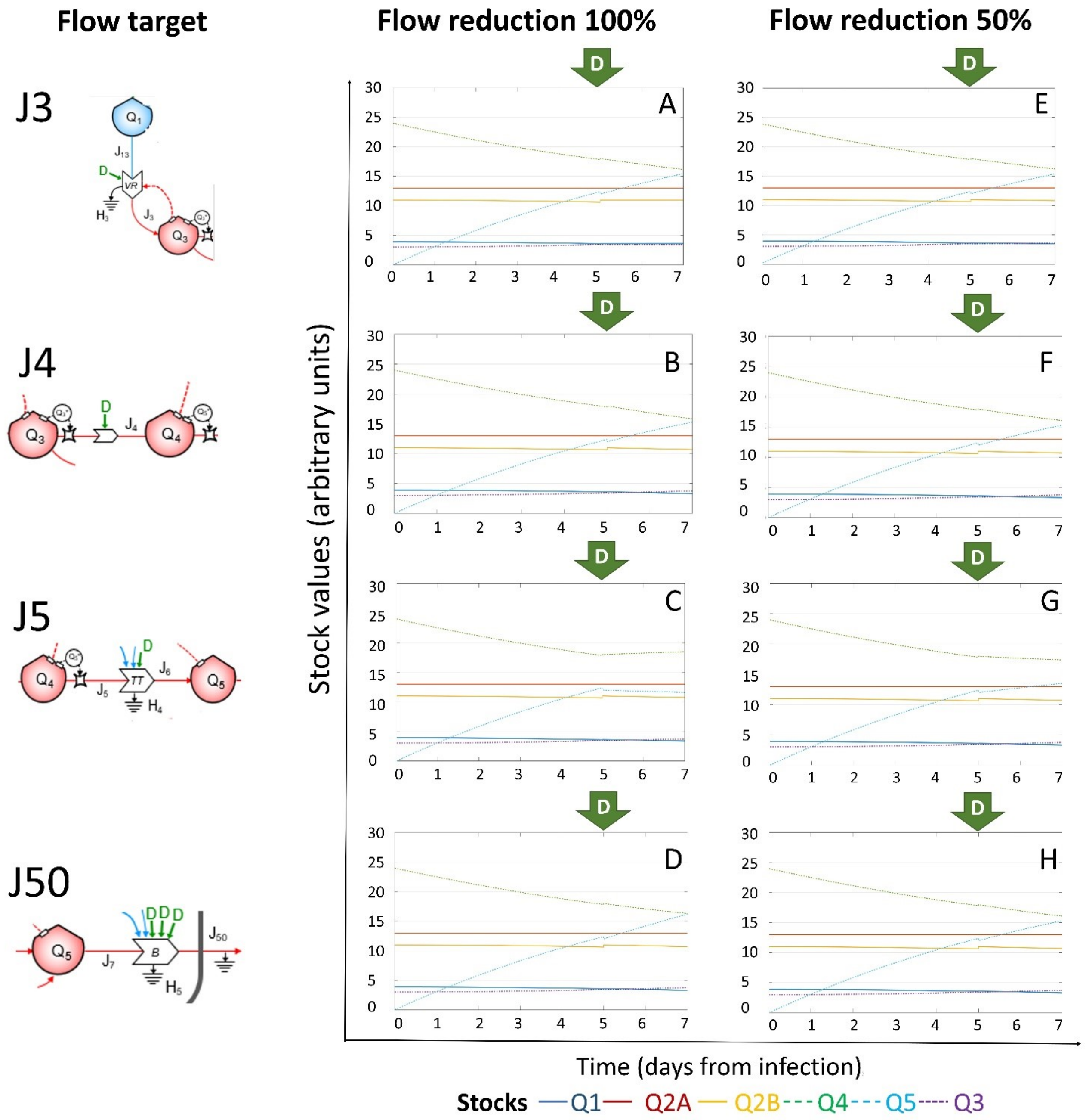

Supplementary Methods: the simulation code

In our set of simulations, we modelled the temporal evolution of state variables (stocks) generated by the inflow and outflow of resources and quantified as embedded energy equivalents (ATP-eq). Scilab (https://www.scilab.org), an open-source scientific computation software, was used for such a purpose.

The architecture of our simulator is composed by three files, containing different scripts: CV_MainConsole, contains the main instruction for compiling the simulation, as well as the initial conditions. The file CV_system contains the set of differential equations, while CV_initiation contains the input equation, that describes the energy inflow to the system. The main file (CV_MainConsole) is contained in a folder, while the other two files are contained in a sub-folder, named ‘model’.

Data obtained from each simulation run, in form of files, with .cvs extension, are used to plot the results.

Supplementary Methods Table 2

Code description and Script

File name	Script
CV_MainConsole	//Testing the COVID-19 model using literature data<br>clc<br><br>//Import CV functions<br>getd('model');<br><br>//Setting CV model parameter<br>//Stocks initial values refer to viral charge equivalent to 10k virions<br>//Parameters k refer to literature values<br><br>param = [];<br>param.k0 = 3.9e-6;<br>param.k1 = 6.9e-5;<br>param.k2A = 2.6e-5;<br>param.k2B = 1.6e-5;<br>param.k3 = 1e-1;<br>param.k4 = 6.9e-5;<br>param.k5 = 1.7e-3;<br>param.k6 = 3e-3;<br>param.k7 = 8.3e-4;<br>param.k13 = 8.3e-2;<br>param.k15 = 1.7e-3;<br>param.k17 = 8.3e-4;<br>param.k20A = 3.9e-6;<br>param.k20B = 1.6e-6;<br>param.k21A = 3.9e-6;<br>param.k21B = 3.9e-6;<br>param.k23 = 8.3e-2;<br>param.k25 = 1.7e-3;<br>param.k27 = 1.7e-3;<br>param.k35 = 4.6e-5;<br>param.k50 = 2e-3;<br>param.J = 0;<br>param.R = 1.7e2;<br>param.E = 0;<br>param.H = 0;<br><br>//Setting initial conditions (TBD)<br>//Time start, end and steps resolution<br>Tbegin = 0;<br>Tend = 7;<br>Tstep = 1/86400<br><br>//Initial condition for stocks<br>Q1_0 = 3.9e0;<br>Q2A_0 = 1.3e1;<br>Q2B_0 = 1.1e1;<br>Q3_0 = 3;<br>Q4_0 = 2.4e-2;<br>Q5_0 = 0;

<table>
<tr><th>File name</th><th>Script</th></tr>
<tr><td></td><td><pre>//Assigning ODE solver data
y0 = [Q1_0;Q2A_0;Q2B_0;Q3_0;Q4_0;Q5_0];
t0 = Tbegin;
t = Tbegin:Tstep:(Tend+100*%eps);
f = CV_system

//Solving the system
CV = ode(y0, t0, t, f);</pre></td></tr>
<tr><td>CV_system</td><td><pre>//set of ODEs for our COVID-19 model
function CVdot=CV_system(t, CV, param)

//fetching parameters
k0 = param.k0;
k1 = param.k1;
k2A = param.k2A;
k2B = param.k2B;
k3 = param.k3;
k4 = param.k4;
k5 = param.k5;
k6 = param.k6;
k7 = param.k7;
k13 = param.k13;
k15 = param.k15;
k17 = param.k17;
k20A = param.k20A;
k20B = param.k20B;
k21A = param.k21A;
k21B = param.k21B;
k23 = param.k23;
k25 = param.k25;
k27 = param.k27;
k35 = param.k35;
k50 = param.k50;
R = param.R;

//Fetching solutions

Q1 = CV(1,:);
Q2A = CV(2,:);
Q2B = CV(3,:);
Q3 = CV(4,:);
Q4 = CV(5,:);
Q5 = CV(6,:);

//Evaluation of initiation

I = CV_initiation(param);
funcprot(0)

//Compute CVdot</pre></td></tr>
</table>

File name	Script
	//If replication is not enough, no outflow as virion<br><br>if Q3 < 3e-2 then k4 = 0<br>end<br><br>//If virions are not enough, no outflow as virion<br><br>if Q4 < 2.4e-4 then k5 = 0<br>end<br><br>//Computation<br><br>Q1dot = ((I)*(1+Q2A))+(k21A*Q2A+k21B*Q2B) - (k1*Q1) - (k13*Q1*Q2B*Q3*Q5) - (k15*Q1*Q2B*Q4) - (k17*Q1*Q2B*Q5)<br>Q2Adot = (k2A*Q1) - (k21A*Q2A) - (k20A*Q2A)<br><br>Q2Bdot = (k2B*Q1*(1+Q4)) - (k21B*Q2B) - (k23*Q1*Q2B*Q3*Q5) - (k25*Q1*Q2B*Q4) - (k27*Q1*Q2B*Q5) - (k20B*Q2B)<br><br>Q3dot = (k3*Q1*Q2B*Q3*Q5) - (k4*Q3)<br><br>Q4dot = (k4*Q3) - (k5*Q1*Q2B*Q4) - (k35*Q4)<br><br>Q5dot = (k6*Q1*Q2B*Q4) - (k7*Q1*Q2B*Q5)+(k35*Q4)<br><br>CVdot = [Q1dot; Q2Adot; Q2Bdot; Q3dot; Q4dot; Q5dot];<br><br>endfunction
CV_initiation	//Computes the initiation function for CV energetics<br>//Initiation function refers to the energy flow input to Q1<br><br>function I=CV_initiation(Q1, Q2A, Q2B, Q3, Q4, Q5, param)<br><br>//It returns the initiation for the CV model<br>//Fetching<br><br>k0 = param.k0<br>R = param.R<br><br>//Define the function I<br><br>I = (k0*R);
